# Effects of maternal appeasing substance and castration status on growth performance and health in newly received, high-risk beef calves

**DOI:** 10.1093/tas/txag038

**Published:** 2026-03-27

**Authors:** Robin A Cheek, Elle Johnston, Rosie Whittle, Jana Reynolds, Doug Galloway, Elizabeth B Kegley, Jeremy G Powell, Shawna Weimer

**Affiliations:** Department of Animal Science, University of Arkansas System Division of Agriculture, Fayetteville, AR, 72701, United States; Department of Poultry Science, University of Arkansas System Division of Agriculture, Fayetteville, AR, 72701, United States; Department of Poultry Science, University of Arkansas System Division of Agriculture, Fayetteville, AR, 72701, United States; Department of Animal Science, University of Arkansas System Division of Agriculture, Fayetteville, AR, 72701, United States; Department of Animal Science, University of Arkansas System Division of Agriculture, Fayetteville, AR, 72701, United States; Department of Animal Science, University of Arkansas System Division of Agriculture, Fayetteville, AR, 72701, United States; Department of Animal Science, University of Arkansas System Division of Agriculture, Fayetteville, AR, 72701, United States; Department of Poultry Science, University of Arkansas System Division of Agriculture, Fayetteville, AR, 72701, United States

**Keywords:** beef cattle, appeasing substance, receiving

## Abstract

The objective was to determine the efficacy of a maternal bovine appeasing substance (**MBAS**) on growth performance, overall health, and behavioral outcomes. Crossbred calves (*n* = 80; steers at arrival [**SAA**, *n* = 33], recently castrated males [**RCM**, *n* = 47]) were purchased and used in a 42-d receiving study. Calves were stratified by d –1 body weight and arrival castration status (**ACS**), then allocated randomly to 1 of 8 groups (10 calves/group [4 to 5 SAA/group, 5 to 6 RCM/group]). Groups were assigned to 1 of 2 treatments (4 groups/treatment), which included cattle receiving no maternal bovine appeasing substance (**CON**) or cattle that were administered a 2.5 ml dose of FerAppease (FerAppease Bovine, FERA Diagnostics and Biologicals, College Station, TX), a **MBAS** which was applied topically to the nuchal skin on d 0. Calves were housed on 0.45-ha pens, provided ad libitum access to bermudagrass hay, and offered a grain supplement (1.82 kg/d) to meet or exceed nutrient requirements. Body weights were recorded on d 0, 3, 14, 21, 28, 41, and 42 and used to calculate average daily gain. Calves (5 calves/group; *n* = 3 RCM and 2 SAA/group) were selected for sampling on d 0, 3, 28, and 42 for serum haptoglobin concentrations. Accelerometers were placed on 16 calves (*n* = 1 SAA and 1 RCM/group) for 7 days to record activity. Behavior measurements inside the chute included video collections recording each calf in the chute on d 3, 14, 28, and 42, and chute exit velocity (**CEV**) was measured on d 3, 14, 28, 41, and 42. Data were analyzed using MIXED and GLIMIX procedures in SAS 9.4. Body weights, haptoglobin, and behavior data were analyzed as repeated measures. Kenward-Rogers was specified as the degrees of freedom selection, utilizing a diagonal covariant structure. MBAS administration did not affect overall growth performance (*P* ≥ 0.3902) during the 42-d period. However, there was a treatment × ACS interaction (*P* = 0.04) for morbidity, CON RCM had a greater incidence compared to CON SAA, and the MBAS RCM were intermediate and did not differ from the MBAS SAA. There was a tendency for treatment × ACS interaction (*P* = 0.08) for serum haptoglobin concentrations to be greatest for CON RCM. The interactions found between ACS and treatment suggest MBAS could improve health outcomes for recently castrated beef calves. To conclude, MBAS did not affect growth performance but could potentially be used to improve the health of high-risk calves in the following 2 weeks of receiving.

## Teaser

Maternal bovine appeasing substance (MBAS) did not improve growth performance; however, results indicate the potential for MBAS to decrease respiratory morbidity in high-risk beef calves, resulting in a positive effect on health for recently castrated males.

## Introduction

Beef cattle production systems represent about 17% of the $462 billion in total cash receipts for agricultural commodities in the United States (Knight 2022). The health and management of newly received cattle continue to pose significant challenges for both animal welfare and profitability for the beef cattle industry. Increasing public concern over animal welfare in the United States cattle industry has prompted scientists to pursue new management solutions.

Bovine respiratory disease (**BRD**) remains the most economically important disease, which costs producers in North America $800 to $900 million every year despite the widespread use of vaccines and antibiotics (Blakebrough-Hall et al. 2020). It is well known that stressors cattle experience during the receiving period led to immunosuppression and elevated incidence of BRD (Galyean et al. 2022). During receiving, cattle that are newly weaned experience a variety of stressful procedures, which typically include transportation events, castration, vaccination, branding, and abrupt changes in environment and diet (Hulbert and Moisa 2016). Male cattle are castrated to facilitate management with a goal to reduce aggressive and sexual behaviors and to improve meat quality (Appleby 1986; Faulkner et al. 1992). Castration is recognized as a painful procedure in beef production, which can lead to changes in their behavior and physiological stress.

In a more recent review, Galyean et al. (2022) explain the need for novel management strategies to alleviate stress and immunocompromised calves in the receiving period. Recent studies report positive results with administering a maternal bovine appeasing substance (**MBAS**) to mitigate stress and improve overall health outcomes (Colombo et al. 2020; Pickett et al. 2024; Cooke et al. 2025). However, these authors tested different commercial products in their experiments. The MBAS that Colombo et al. (2020) used included a mixture of fatty acids that simulates the composition of the natural bovine appeasing pheromone (Nutricorp; Aras, Brazil). The MBAS that Pickett et al. (2024) used included a proprietary mixture of fatty acids, including palmitic, oleic, and linoleic acids (Fer Appease; FERA Diagnostics and Biologicals; College Station, TX), which is the product in question for the current study. Appeasing products have shown to facilitate cow-calf bonding through maternal odor recognition by the offspring (Cappellozza and Cooke 2022). This MBAS product has been reported to desensitize regions of the amygdala, decreasing threat perception during stressful management procedures (Cappellozza and Cooke 2022).

Colombo et al. (2020) applied MBAS to high-risk calves at feedlot arrival and reported an improvement in health and performance responses during a 45-d receiving period. Pickett et al. (2024) applied MBAS to Angus-influenced, freshly weaned male calves during receiving, which decreased physiological stress markers, reduced mortality, and increased pen-based activity during a 60-d study. In a 2-year study, Cooke et al. (2025) observed that MBAS improved health and performance in cattle placed on feed as yearlings. The first experiment of this study evaluated Angus steers exposed to typical United States feedyard management and reported MBAS increased feed intake, growth performance, and carcass quality. The second experiment investigated *Bos indicus* bulls, which are more susceptible to stress, and were exposed to 96-h road transport and processing upon arrival. Bulls from the second experiment that had received MBAS had greater feed intake and growth performance and decreased morbidity and mortality rates.

Although research has shown some beneficial effects of administering MBAS in receiving beef cattle, authors have indicated the need for additional research to address the physiological stress response, which includes mitigating the negative effects of BRD on growth performance and morbidity. Therefore, the objective of this study was to investigate the effect of administering MBAS at receiving on growth performance, BRD incidence, health and stress markers, and behavior of newly received cattle. The hypothesis was for MBAS to improve the overall performance of high-risk calves within the 42-d backgrounding period.

## Materials and methods

Animal methods were approved by the University of Arkansas Animal Care and Use Committee (Approval #23056). Eighty crossbred male beef calves (steers at arrival [**SAA**, *n* = 33], recently castrated males [**RCM**, *n* = 47]) were obtained from a cooperative producer, who purchased calves in regional sale barns and then shipped cattle to the University of Arkansas Division of Agriculture Beef Cattle Research Facility near Fayetteville, Arkansas, in September 2023. Upon arrival (d—1), cattle were tagged in the left ear with a unique identification number, weighed, ear notched, and housed overnight in a holding pen with access to hay and water. Ear notches were sent for persistent infection with bovine viral diarrhea virus (**PI-BVDV**) testing (Cattle Stats, LLC, Oklahoma City, OK) within 48 h of cattle arrival, with no calves testing positive. The following morning (d 0), calves were administered respiratory (Pyramid 5, Boehringer Ingelheim Vetmedica, Duluth, GA) and clostridial (Covexin 8, Intervet, Inv., Madison, NJ) vaccinations, and dewormed (Ivomec Plus, Boehringer Ingelheim Vetmedica, Duluth, GA). Intact males were castrated by banding (California bander, Inosol Co., LLC El Centro, CA). Calves received booster vaccinations on d 14. All calves were branded with a hot iron on the right hip and were weighed. The body weights recorded on both days (d –1, d 0) were averaged to represent the initial weight (209 ± 17 kg).

### Experimental design

Calves were stratified by d -1-BW and arrival castration status (ACS) and allocated randomly to 1 of 8 groups (10 calves/group; 4 to 5 SAA/group, 5 to 6 RCM/group). Groups were then assigned to 1 of 2 treatments (4 groups/treatment). Treatments consisted of cattle receiving no maternal bovine appeasing substance (**CON**) and cattle that were administered a 2.5 ml dose of FerAppease (FerAppease Bovine, FERA Diagnostics and Biologicals, College Station, TX), a maternal bovine appeasing substance (**MBAS**) applied topically to the nuchal skin and the skin above the muzzle on d 0 during initial processing. Immediately after processing, calves were sorted into groups. The chute was wiped down with Clorox wipes between the CON and MBAS calves to avoid cross-contamination.

Cattle were housed on 0.45-hectare grass pens, fed a grain-grain by-product supplement (Table 1; 98% DM, 21% CP, 12% ash, 68% NDF, 35% ADF), and had libitum access to Bermudagrass hay (96% DM, 13% CP, 9% ash, 72% NDF, 33% ADF). Groups were assigned to pens so that fence-line contact was minimized by the presence of an alleyway between MBAS and CON pens. Bunk readings were evaluated each day to determine when to increase the amount of supplement delivered. Calves were offered a grain supplement at 0.91 kg/d (as-fed basis) on d 0, and this was increased to a maximum of 1.82 kg/d of supplement for the remainder of the 42-d backgrounding trial. When feed bunks were checked each morning, any refusals from the previous day were collected, weighed, and subsamples frozen for later analyses of dry matter (**DM**).

**Table 1:** Ingredient composition of grain supplement (as-fed basis).

Ingredient	%
**Cracked corn**	63.36
**Dried distillers’ grains**	26.00
**Molasses**	2.0
**Limestone**	2.0
**Salt, white**	1.0
**Corn/Rumensin premix^1^**	0.40
**Vitamin A, D, E premix^2^**	0.10
**Ruminant trace mineral premix**	0.09
**Vitamin E premix^3^**	0.05

1 Provides 22 g monensin/kg of premix.

2 Contains 880,000 IU Vitamin A, 1,760,000 IU Vitamin D, and 1100 IU Vitamin E/kg of premix.

3 Contains 44,000 IU/kg premix.

### Data collection

Body weights were recorded initially (d –1 and 0) and before supplement feeding on the mornings of d 3, 14, 21, 28, 41, and 42. Average daily gain (**ADG**) was calculated for the interim and final periods based on the averages of initial and final full body weights that were recorded on the 2 consecutive days.

Cattle were observed daily (0800 h) by trained personnel for signs of bovine respiratory disease (**BRD**) beginning the morning of the day after processing (d 1). Clinical signs of BRD included depression, cough, poor appetite, and respiratory distress. Cattle were given a Clinical Attitude Score (**CAS**) by implementing a 4-point scale (0 = normal, 1 = mild BRD, 2 = moderate BRD, 3 = severe BRD, 4 = moribund) method based on depression, anorexia, respiratory, and temperature (“DART”) as described by Step et al. (2008) and Wilson et al. (2015). The individual checking pens and scoring cattle remained the same for the entirety of the study. Cattle with scores > 0 were brought to the chute, where a rectal temperature was taken. Cattle with a CAS ≥ 1 and a rectal temperature ≥ 40°C were treated according to a preplanned antibiotic protocol. The BRD Therapy 1 (Nuflor, Merck Animal Health, Rahway, NJ) was administered at 6 mL/45.45 kg BW subcutaneously in the neck. Calves receiving BRD Therapy 1 were sent back to their home pen. If the calf scored a CAS ≥ 1 following BRD Therapy 1 and if rectal temperature was ≥ 40°C, calves received BRD Therapy 2 (Baytril, Elanco Animal Health, Shawnee, KS) at a rate of 5.7 mL/45.45 kg BW subcutaneously in the neck and were sent back to their home pen. If rectal temperature was ≥ 40°C at the time of reevaluation, the calf would receive BRD Therapy 3 (Excenel, Zoetis, Florham Park, NJ) administered as a 2 mL/45.45 kg BW dosage subcutaneously in the neck for 3 consecutive days. During the 3 days, the calf would be placed in a hospital pen to be 14 monitored. After 3 days, if the calf remained in the same state of health and rectal temperature was ≥ 40°C, calves received a final BRD Therapy 4 (Draxxin, Zoetis) dosed at 1.1 mL/45.45 kg BW subcutaneously in the neck. After administering BRD therapy 4, if the CAS was ≥ 2 and the rectal temperature was ≥ 40°C, then the calf was considered nonresponsive, and no further treatments were given. If BRD symptoms were present > 21 days after administering the previous therapy, symptoms were considered a new BRD episode, and treatment began with BRD Therapy 1. Records were kept of all calves pulled from each pen, their CAS, rectal temperatures, all antibiotics administered, and medication costs. If a calf was treated ≥ 3 times with antibiotics and failed to gain > 0.45 kg/d for the 42-d period, then it was considered and deemed as a chronic calf. If a calf did not live for the duration of the 42-d trial, this was recorded, and the calf was necropsied to determine the cause of death. One RCM that was on the MBAS treatment died and was removed from all data analyses.

Upon exiting the working chute, exit velocities (m/s) for each calf were recorded on a Polaris Multi-Event Timer on d 3, 14, 28, 41, and 42. Chute exit velocity measurements could not be collected for d 0 due to technical difficulties. The timer beams were 0.61 m high and placed 1.83 m from the front of the chute on the left side. As cattle came through the chute, the tag number and velocity were recorded.

On d 0, 3, 28, and 42, a subset of calves (5 calves/group; *n* = 16 SSA, *n* = 24 RCM) were bled via jugular venipuncture to evaluate serum haptoglobin concentrations. Blood was drawn with a 26-gauge needle into vacuum tubes containing a clot-activating gel (BD Inc., Franklin Lakes, NJ). Blood samples were allowed to sit at room temperature for at least 30 min to allow clot formation, centrifuged at 2,060 x g for 20 min, and serum was extracted and stored at −80°C until analysis. Serum concentrations of haptoglobin were measured with a commercial ELISA (Kit # E-10HPT: Immunology Consultants Laboratory Inc., Portland, OR).

On d 0, a subset of calves (2 calves [*n* = 8 SAA, *n* = 8 RCM] out of the original 5 calves/group) were outfitted with triaxial accelerometers (HOBO Pendant G Acceleration Logger, Onset Computer Corporation, Bourne, MA). The devices were placed on the afternoon of d 0 and removed on the morning of d 14. The accelerometers were attached to the left pastern with zip ties and wrapped with VetRap for stability (Finney et al. 2018). Once the accelerometers were removed, the recordings were uploaded into an Excel spreadsheet and later analyzed. It is important to note that only 15 out of the 16 accelerometers recorded data for this period, and data are reported for d 0 to 7. Data were extracted from the accelerometers as coordinates at 20 second intervals. Raw accelerometer data were processed in R (v4.4.1) and RStudio (v.2024.04.02 Build 764) according to methods described in Brown et al. (2015). Briefly, 2-dimensional accelerometer data (X, Y) from each animal were clustered through medoid portioning using clustering large applications (CLARA; *k = *3) to identify 3 distinct behavioral groups: standing, lying on sternum, and lying with leg out. Daily counts, bout frequency, and mean bout duration for each cluster were computed per animal and treatment and exported for downstream analyses. For this analysis, a bout represented each time cattle changed positions, bout duration was the amount of time the cattle spent in each position, and the proportion of time was the total time spent in each position.

Video recordings were collected for each calf in the chute on d 3, 14, 28, and 42 using GoPro Hero 8 cameras (San Mateo, CA, USA) for behavior observations. Recording equipment was set up before the beginning of each collection day. The cameras were placed in 2 different locations: (i) a camera was placed on a tripod in front of the chute exit event timer, and (ii) a camera was hung above the chute, off to the right side, directly looking down on the chute. The video recordings were divided into 17 min, 28 s segments. Each video was viewed to capture and record the tag number and time each calf entered and exited the chute, and each calf was observed for 7 behaviors: front end down, head tossing, nasal discharge or salivation, vocalizing, white eyes visible, and jumping/thrashing/pushing (Table S1). The chute score was determined based on a score from 1 to 5 (Table S2; 1 = no movement, 5 = rearing, twisting, and violently struggling). The video was observed a second time to determine the subjective chute score based on the intensity and number of previously recorded behaviors.

### Statistical analysis

Data were analyzed as a generalized complete block design with calf identified as the experimental unit. All data were analyzed using MIXED and GLIMMIX procedures of SAS 9.4 (SAS Inst. Inc., Cary, NC). Body weights, haptoglobin concentrations, chute exit velocity measures, and accelerometer data were analyzed as repeated measures. This MIXED procedure included ACS, treatment, day, and replication (group within treatment) in the class statement. For all MIXED models, replication was considered a random effect with the individual calf as the subject. Haptoglobin concentrations were transformed using a logarithmic transformation to achieve a normal distribution of data points. For models analyzed as repeated measures, Kenward-Rogers was specified as the degrees of freedom selection, utilizing a diagonal covariant structure. The model tested for differences in treatment, day, ACS, and all possible interactions between the 3.

Behavior in the chute, ADG, and morbidity data were analyzed using MIXED procedures, which included treatment, replication, and ACS in the class statement. This model tested for differences in treatment, ACS, and for an interaction of treatment × ACS. Binomial data included morbidity and chute behavior and were analyzed as percentages using GLIMMIX procedures for the effects of treatment, ACS, and an interaction of treatment × ACS. MIXED and GLIMMIX procedures for behavior, ADG, and morbidity data specified Kenward-Rogers as the degrees of freedom selection method.

Significance was declared when *P* ≤ 0.05, with tendencies declared when *P* > 0.05 and ≤ 0.10. If there were any interactions that were significant (*P* ≤ 0.05), treatment and ACS means were separated using a t-test using the PDIFF option in SAS.

## Results

### Growth performance

**Figure 1:**
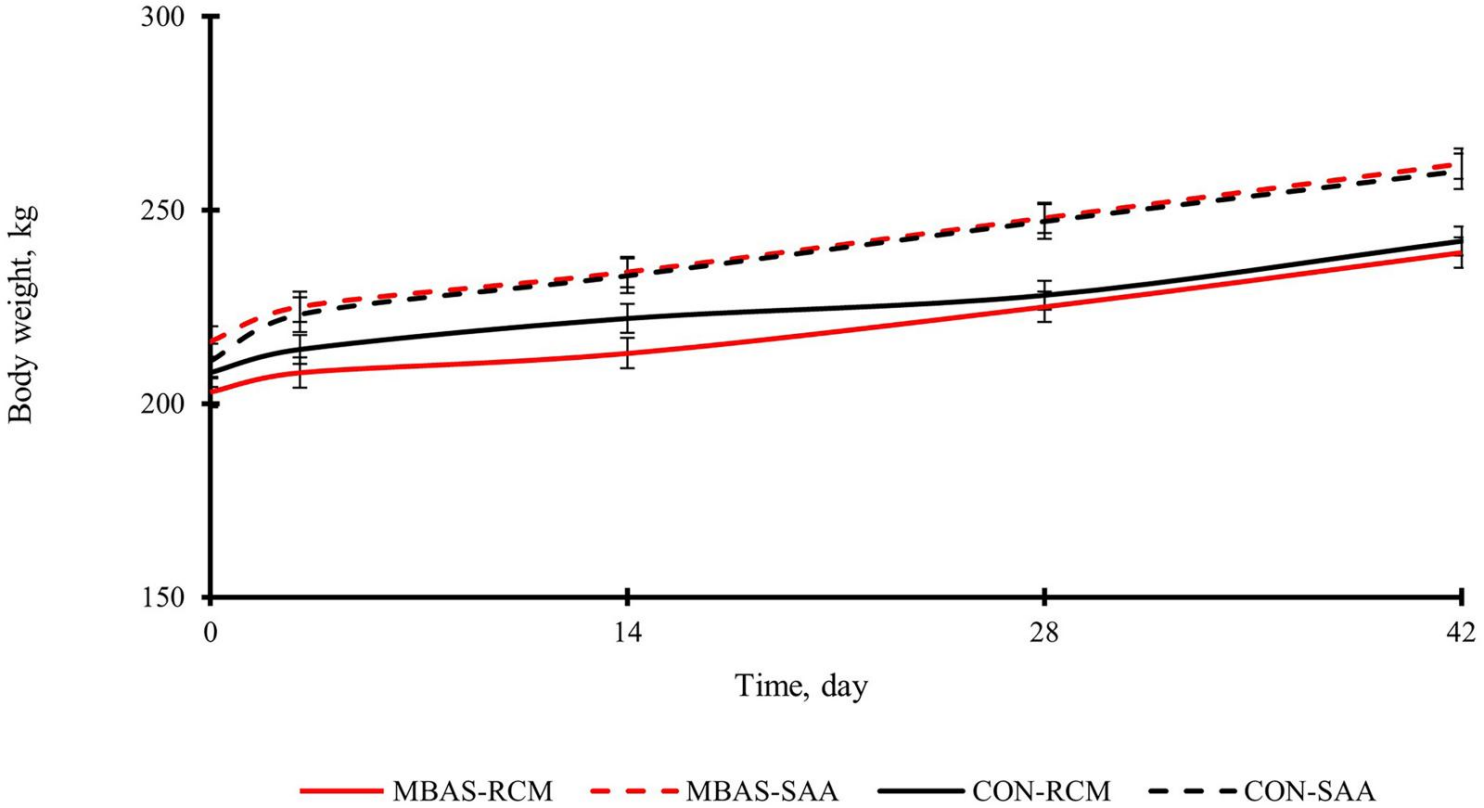
Effects of MBAS and arrival castration status (ACS) on body weights during a 42-d trial. Treatment (*P* = 0.3902); Day (*P* < 0.0001); ACS (*P* < 0.0001); Treatment × day (*P* = 0.9682); Treatment × ACS (*P* = 0.0656); Treatment × ACS × day (*P* = 0.5469).

Initial body weights (Fig. 1) were similar (*P* = 0.9666) for both treatment groups (210 ± 18 kg). There was a tendency for an interaction of treatment and ACS (*P* = 0.0656) for the overall body weights recorded over the 42-d period to be lower for the RCM administered MBAS. There were no main effects of MBAS administration on body weights (*P* ≥ 0.3902), but there was a main effect of ACS (*P* < 0.0001). Body weights were greater for SAA (213 ± 18 kg) versus RCM on d 0 (206 ± 17 kg), regardless of treatment or day (*P* < 0.0001). There were no main effects of treatment (*P* = 0.9473) or any interactions of treatment and ACS (*P* = 0.2740) for overall ADG (Table 2), but there was a tendency between d 14 and d 28 (*P* = 0.0552) for MBAS to increase the rate of ADG in RCM. However, there was a main effect of treatment on ADG during the first 2 weeks (*P* = 0.0238) of the receiving period, which revealed calves that did not receive MBAS administration had greater ADG. There was a main effect of ACS on the ADG (*P* < 0.0001), SAA gained at a faster rate in comparison to RCM calves.

**Table 2:** Effect of maternal bovine appeasing substance (MBAS) and arrival castration status (ACS) on growth average daily gain (ADG) of newly received calves over a 42-d period.

Item	MBAS		CON		SEM	*P*-value		
	RCM**^1^**	SAA**^2^**	RCM	SAA		ACS	Treatment	ACS × Treatment
	*n* = 23	*n* = 16	*n* = 24	*n* = 16				
**ADG^3^, kg/d**								
**d 0 to d 3**	1.52	2.89	1.94	3.77	0.41	< 0.0001	0.0839	0.5393
**d 3 to d 14**	0.50	0.89	0.76	0.99	0.16	0.0371	0.2380	0.5879
**d 0 to d 14**	0.72	1.32	1.01	1.58	0.13	< 0.0001	0.0238	0.9106
**d 14 to d 28**	0.80^a^	0.95^a^	0.43^b^	0.97^a^	0.11	0.0010	0.0827	0.0552
**d 0 to d 28**	0.76	1.14	0.72	1.23	0.08	< 0.0001	0.4967	0.2067
**d 28 to d 42**	1.05	1.00	0.97	0.90	0.10	0.4950	0.3307	0.8981
**d 0 to d 42**	0.86	1.09	0.80	1.15	0.06	< 0.0001	0.9473	0.2740

1 RCM = recently castrated males.

2 SAA = Steers at arrival.

3 Means within ACS and treatment without a common superscript differ (*P* ≤ 0.05).

### Morbidity

There was an interaction of treatment and ACS (*P* = 0.0448) on the percentage of cattle that were treated at least once with antibiotics, RCM that did not receive MBAS on d 0 had the greatest incidence of BRD (Table 3). Further, there was a tendency for an ACS × treatment interaction (*P* = 0.0988) for the average total number of antibiotic treatments per calf to be the greatest for RCM on the CON treatment. There were no main effects of MBAS administration (*P* ≥ 0.2080) for morbidity associated with BRD. There was a main effect of ACS found for the percentage of cattle treated at least once (*P* = 0.0064) and the number of antibiotic treatments administered per calf (*P* = 0.0018), with the greatest values for RCM from both treatments. The day on which calves were treated for BRD (*P* ≥ 0.6010), or rectal temperatures (*P* ≥ 0.2080), were not affected by MBAS treatment or ACS. The statistical model did not converge due to low observations for the percentage of cattle treated twice or thrice. There were only 3 RCM (MBAS, *n* = 1; CON, *n* = 2) treated twice and 1 RCM (MBAS, *n* = 1) treated thrice.

**Table 3:** Effect of maternal bovine appeasing substance (MBAS) and arrival castration status (ACS) on morbidity incidence of BRD of newly received calves over a 42-d period.

Item	MBAS		CON		SEM	*P*-value		
	RCM**^1^**	SAA**^2^**	RCM	SAA		ACS	Treatment	ACS × Treatment
	*n* = 23	*n* = 16	*n* = 24	*n* = 16				
**Cattle treated at least once^3^, %**	40.88^ba^	29.37^ba^	62.53^a^	6.24^b^	11.33	0.0064	0.4768	0.0448
**Day 1^st^ treated**	5	4	6	3	4	0.6010	0.9561	0.7568
**Rectal temperature of 1^st^ treated, ˚C**	40.5	40.8	40.4	40.2	0.78	0.8204	0.2080	0.4343
**Cattle treated at least twice^4^, %**	–	–	–	–	–	–	–	–
**Cattle treated at least thrice^5^, %**	–	–	–	–	–	–	–	–
**Number of antibiotic treatments per calf^3^**	0.50^ba^	0.29^ba^	0.71^a^	0.06^b^	0.14	0.0018	0.9297	0.0988

1 RCM = recently castrated males.

2 SAA = Steers at arrival.

3 Means within ACS and treatment without a common superscript differ (*P* ≤ 0.05).

4 GLIMMIX model did not converge: MBAS (RCM, *n* = 1), CON (RCM, *n* = 2).

5 GLIMMIX model did not converge: MBAS (RCM, *n* = 1).

### Haptoglobin concentrations

**Figure 2:**
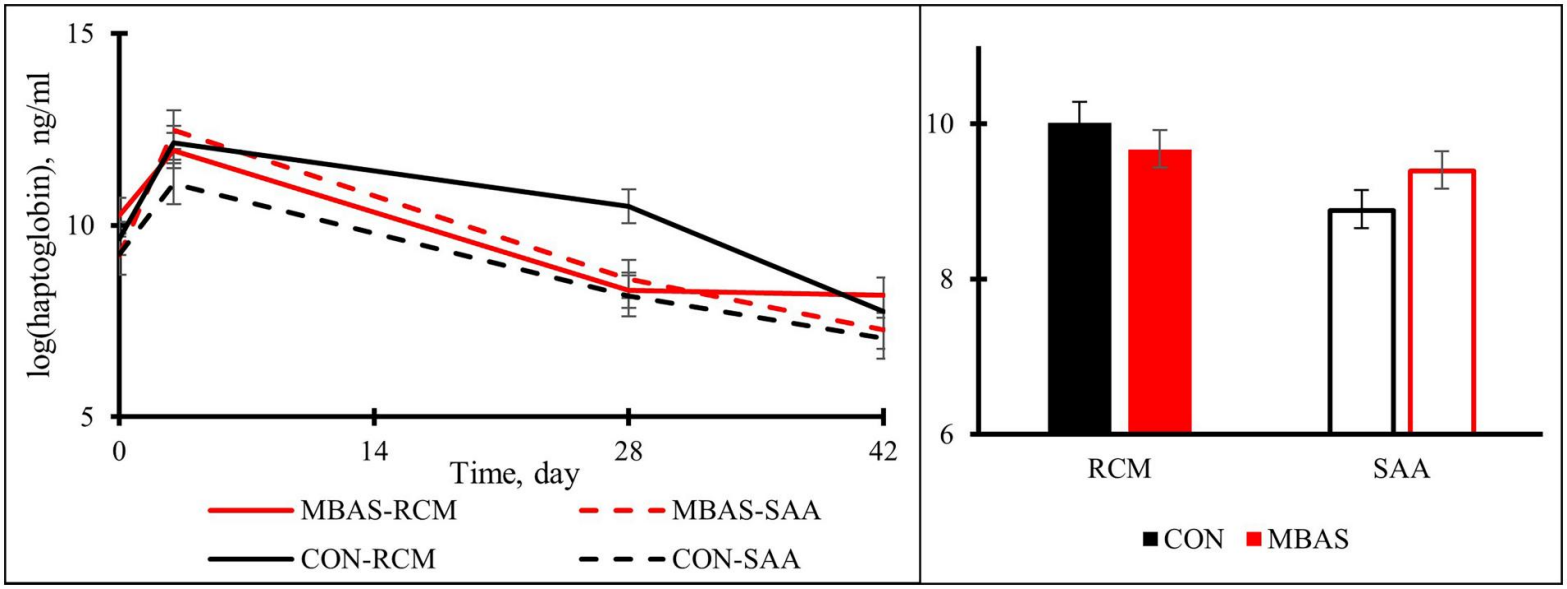
Effects of MBAS and ACS on serum haptoglobin concentrations during a 42-d trial. Panel A (left): Treatment (*P* = 0.7298); Day (*P* < 0.0001); ACS (*P* = 0.0043); Panel B (right): Treatment × day (*P* = 0.1437); Treatment × ACS (*P* = 0.0801); Treatment × ACS × day (*P* = 0.1946).

There was a tendency for a treatment × ACS interaction (*P* = 0.0801) for serum haptoglobin concentrations to be greater for RCM calves on the CON treatment (Fig. 2). There was a main effect of ACS (*P* = 0.0043), RCM had greater serum haptoglobin concentrations than SAA during the receiving period. Haptoglobin concentrations were the greatest for all calves on d 3, revealing a day effect (*P* < 0.0001), and concentrations decreased to similar concentrations for the remainder of the study. The results showed no main effects of MBAS administration (*P* = 0.7298), treatment × day (*P* = 0.1437), or any 3-way interactions (*P* = 0.1946).

### Chute exit velocity

**Figure 3:**
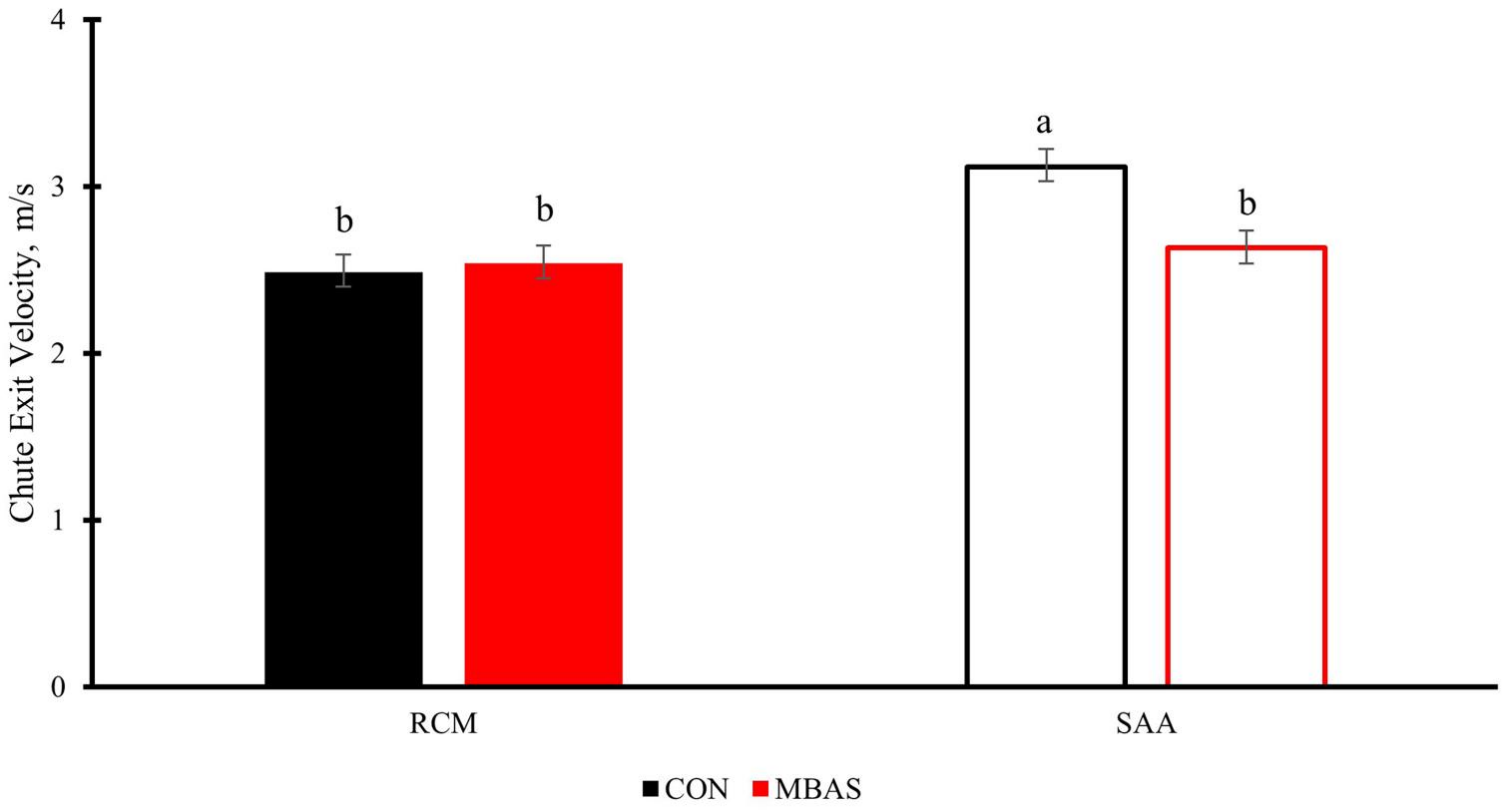
Effects of MBAS and ACS on chute exit velocity (m/s) across all days during a 42-d trial. Treatment (*P* = 0.0282); Day (*P* < 0.0001); ACS (*P* = 0.0003); Treatment × day (*P* = 0.5458); Treatment × ACS (*P* = 0.0060); Treatment × ACS × day (*P* = 0.1143). Means within ACS without a common superscript differ (*P* ≤ 0.05).

There was a treatment × ACS interaction (Fig. 3; *P* = 0.0060) for CEV, where CON-SAA had the greatest CEV (3.11 m/s) compared to SAA that were administered MBAS (2.62 m/s). There was a trend for a potential 3-way interaction (*P* = 0.1143) of treatment, ACS, and day for CEV, which revealed main effects of treatment (*P* = 0.0282), day (*P* < 0.0001), and ACS (*P* = 0.0003). The main effect of treatment showed that the CEV was slower for calves that received MBAS (2.59 m/s) than CON (2.80 m/s).

### Behavior observed via accelerometers

**Figure 4:**
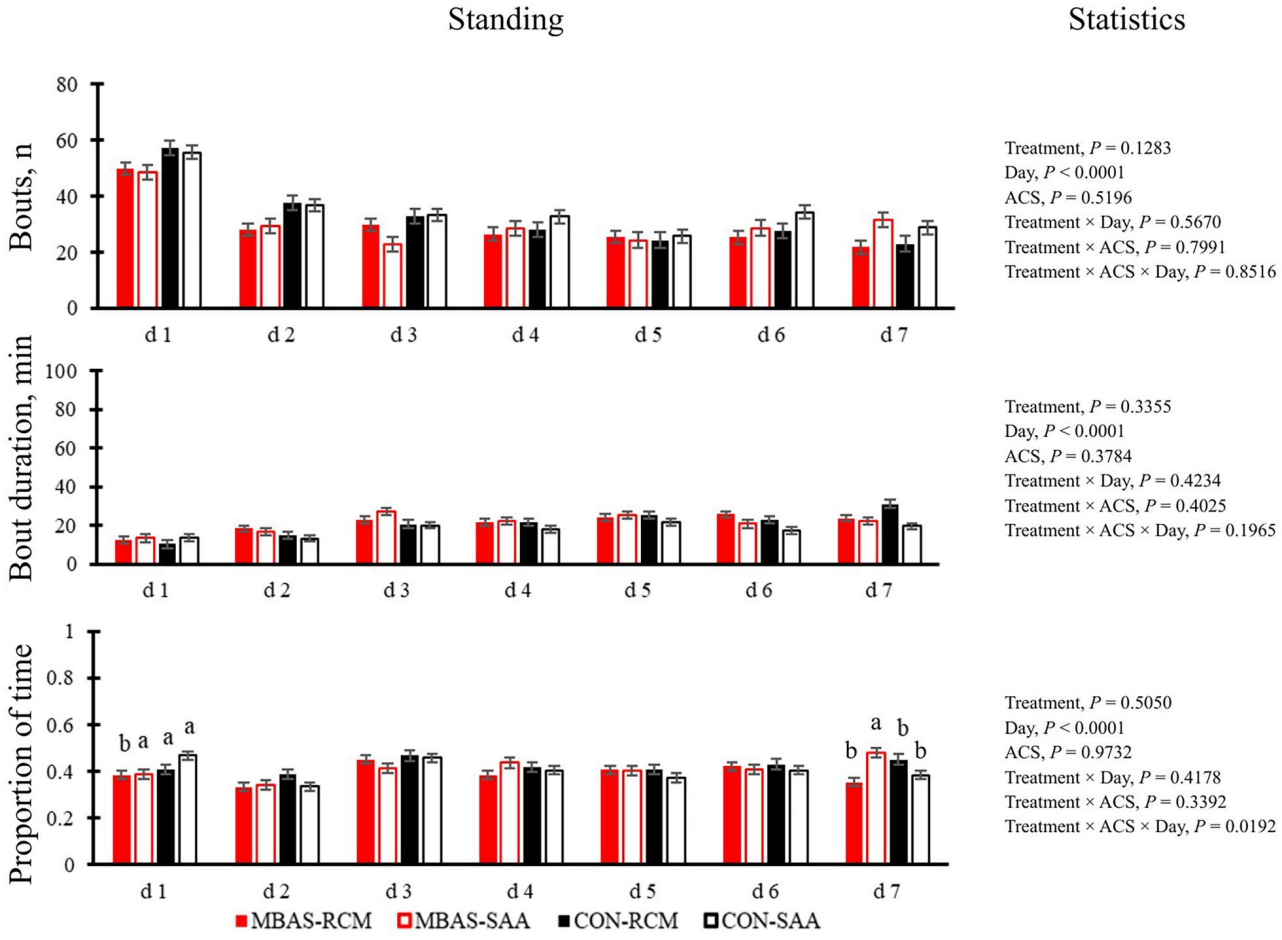
Effect of maternal bovine appeasing substance (MBAS) and arrival castration status (ACS) on number of bouts, bout duration (min), and proportion of time spent standing during a 7-d period. Means within a day without a common superscript differ (*P* ≤ 0.05).

**Figure 5:**
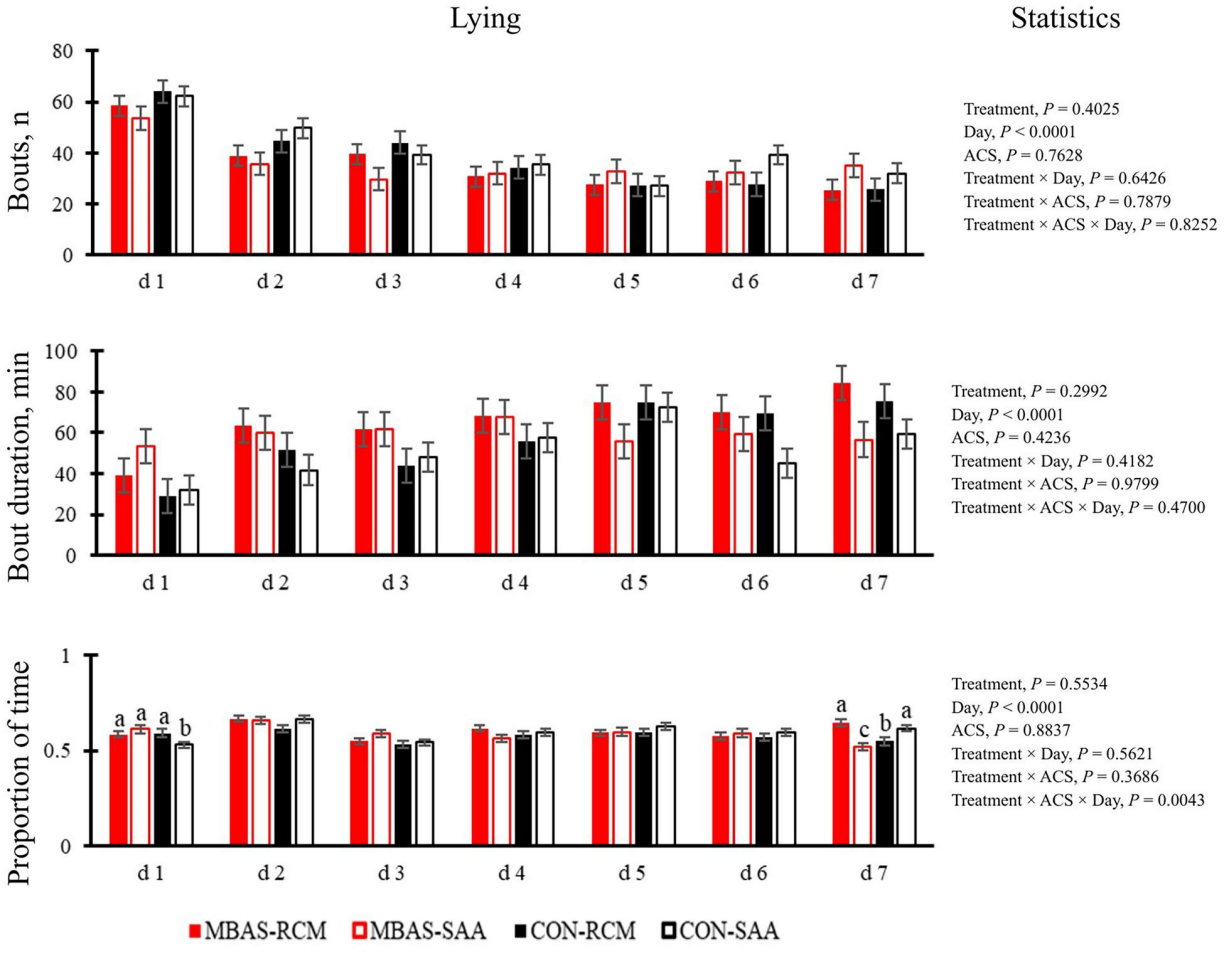
Effect of maternal bovine appeasing substance (MBAS) and arrival castration status (ACS) on number of bouts, bout duration (min), and proportion of time spent lying during a 7-d period. Means within a day without a common superscript differ (*P* ≤ 0.05).

The effect of MBAS and ACS on cattle behavior outside of the chute system included the number of bouts, bout duration (min), and proportion of time spent standing (Fig. 4) versus lying (Fig. 5).

There was a 3-way interaction of treatment, ACS, and day (*P* = 0.0192) on the proportion of time cattle spent standing during the 7-d period. On d 1 SAA on the CON treatment spent the greatest proportion of time standing versus the RCM on the MBAS treatment (*P* = 0.0274). On d 7 RCM on MBAS (*P* = 0.0035) and SAA on the CON (*P* = 0.0227) treatment spent the least proportion of time standing. There were no other interactions found for the number of bouts or bout duration (*P* ≥ 0.1965). There were day effects found for the number of bouts, bout duration, and proportion of time spent standing (*P* < 0.0001). The day effects revealed that the number of bouts decreased similarly, and the bout duration slightly increased for all calves over the 7-day period.

There was a 3-way interaction of treatment, ACS, and day (*P* = 0.0043) on the proportion of time cattle spent lying during the 7-d period. Complementary to the standing results, there was a difference found on d 1 where SAA on the CON spent the least proportion of time lying (*P* = 0.0425). On d 7 RCM on the MBAS (*P* = 0.0021) and SAA on the CON (*P* = 0.0151) treatment spent the greatest proportion of time lying. There were no other interactions found for the number of bouts or bout duration (*P* ≥ 0.4182). Day effects were found for the number of bouts, bout duration, and the proportion of time spent lying (*P* < 0.0001). The day effects demonstrated that the number of bouts decreased while the bout duration increased over the 7-d period.

### Behavioral observations in the chute

Chute score data are provided within Table S3. Chute scores were not influenced by treatment (*P* ≥ 0.2750; CO*N* = 2.44, MBAS = 2.41) but were affected by ACS on d 3 (*P* = 0.0126; RCM = 2.29, SAA = 2.82) and d 14 (*P* = 0.0413; RCM = 1.93, SAA = 2.31). There were no interactions of treatment and ACS (*P* = 0.9491) or treatment, ACS, and day (*P* = 0.4029) found on chute scores. Overall chute scores were 2.42 for all calves. All other behavior observations within the chute system are reported in Table 4. There were only main effects of ACS found on the behavior within the chute system. The percentage of cattle that were observed head-tossing was greater for SAA on d 14 when compared to RCM (*P* = 0.0358). Head tossing percentage tended to be greater (*P* = 0.0861) for CON-RCM than cattle on the MBAS treatment on d 28. Nasal discharge or salivation tended to be greater for SAA regardless of treatment (*P* = 0.0902). The percentage of calves noted for vocalizing or having the whites of their eyes visible was not influenced by ACS (*P* ≥ 0.1351). The percentage of cattle that were jumping, thrashing, or pushing was significantly greater for SAA than RCM on d 42 (*P* = 0.0155). There were no main effects of treatment (*P* ≥ 0.1251) or interactions of treatment and ACS (*P* ≥ 0.1664) found on the behaviors recorded within the chute system.

**Table 4:** Effect of maternal bovine appeasing substance (MBAS) and arrival castration status (ACS) on behavior in the chute of beef calves during a 42-d receiving study.

Item	MBAS		CON		ACS		Treatment		ACS × Treatment	
	RCM**^1^**	SAA**^2^**	RCM	SAA	SEM	*P-*value	SEM	*P-*value	SEM	*P-*value
**Head tossing, %**										
**d 3**	86	94	71	81	6.3	0.3057	7.2	0.1251	10.0	0.8134
**d 14**	68	88	62	87	7.2	0.0358	7.7	0.7994	10.2	0.8847
**d 28**	50	65	75	50	8.9	0.6171	8.3	0.6171	12.9	0.0861
**d 42^3^**	–	–	–	–	–	–	–	–	–	–
**Nasal discharge/salivation, %**										
**d 3**	50	71	67	56	8.7	0.6584	8.3	0.9437	12.8	0.1815
**d 14**	27	53	50	44	9.0	0.3834	8.3	0.5287	12.8	0.1664
**d 28**	41	65	46	62	8.6	0.0902	8.5	0.9131	12.4	0.7593
**d 42**	23	6	12	12	5.8	0.3167	6.1	0.9466	9.2	0.3167
**Vocalizing, %**										
**d 3**	23	23	25	19	7.3	0.7765	6.9	0.8863	10.5	0.7164
**d 14**	23	18	8	19	6.9	0.6392	6.7	0.4018	10.0	0.3417
**d 28^4^**	–	–	–	–	–	–	–	–	–	–
**d 42^5^**	–	–	–	–	–	–	–	–	–	–
**Whites of eyes visible, %**										
**d 3**	9	29	17	19	7.7	0.2213	6.6	0.9373	11.4	0.3164
**d 14**	14	23	12	31	8.0	0.1351	6.7	0.8102	12.0	0.6856
**d 28**	4	23	17	19	7.3	0.1768	6.3	0.4392	10.6	0.2461
**d 42**	9	18	12	25	7.4	0.2285	6.4	0.5486	11.0	0.9482
**Jumping/thrashing/pushing, %**										
**d 3^6^**	–	–	–	–	–	–	–	–	–	–
**d 14^7^**	–	–	–	–	–	–	–	–	–	–
**d 28**	86	83	96	94	5.9	0.6742	6	0.1614	9.5	0.9445
**d 42**	45	71	29	62	8.5	0.0155	8.7	0.2844	12.5	0.7324

1 RCM = recently castrated males.

2 SAA = Steers at arrival.

3 GLIMMIX model did not converge: MBAS-RCM (*n* = 14), MBAS-SAA (*n* = 15), CON-RCM (*n* = 18), CON-SAA (*n* = 16).

4 GLIMMIX model did not converge: MBAS-RCM (*n* = 2), MBAS-SAA (*n* = 2), CON-RCM (*n* = 0), CON-SAA (*n* = 0).

5 GLIMMIX model did not converge: MBAS-RCM (*n* = 0), MBAS-SAA (*n* = 0), CON-RCM (*n* = 0), CON-SAA (*n* = 0).

6 GLIMMIX model did not converge: MBAS-RCM (*n* = 21), MBAS-SAA (*n* = 16), CON-RCM (*n* = 20), CON-SAA (*n* = 16).

7 GLIMMIX model did not converge: MBAS-RCM (*n* = 20), MBAS-SAA (*n* = 16), CON-RCM (*n* = 23), CON-SAA (*n* = 16).

## Discussion

The primary goal of the current experiment was to assess the efficacy of MBAS with consideration of ACS (SAA or RCM) on growth performance, morbidity incidence associated with BRD, cattle behavior, and physiological response. Cattle were newly weaned, lightweight, underwent transportation, and were exposed to a new environment, which made them at high risk for developing BRD according to the description provided by Wilson et al. (2017). While the concept of this experiment was not novel, the behavioral and physiological measurements taken in this 42-d study add to the body of knowledge previously reported in literature. Previous studies (Colombo et al. 2020; Cooke et al. 2020; Pickett et al. 2024) have reported positive effects of MBAS at the receiving phase of the feedlot and in preconditioning programs. Mammals have a mechanism to secrete pheromone molecules, like MBAS, which are released into the atmosphere and detected by target organs. The behavioral mechanism to detect pheromones is termed the “Flehmen Reflex” and is described as the animal elevating and extending its head, retracting the superior lip, and exposing the ear-jaw articulation, for the inhalation of a specific substance (Crowell-Davis et al. 1985). The synthetic analog, MBAS, used in this study was expected to have calming effects, improving cattle welfare and productivity following stressful events.

The results from the current study had a great number of interactions between treatment and ACS, which demonstrates the effect that MBAS had on calves that were castrated at processing and calves that arrived as steers. Banding, used as the method of castration in this experiment, is done by placing a rubber ring on the neck of the scrotum to stop the blood supply to the testicles. This is known to result in chronic ischemia and coagulation necrosis until detachment of the scrotum. The duration of this process is typically 35 to 65 d, which in this study would take place in the entire 42-d trial.

There were no consistent effects of MBAS on growth performance within the receiving period in the current experiment. In turn, Pickett et al. (2024) reported that feedlot steers administered MBAS had greater ADG and final body weights. While the current study showed no consistent treatment effects, the results tended to show the effect of MBAS administration between SAA and RCM. The SAA calves had greater body weights for the entirety of the trial. In turn, the growth performance of RCM was lower over time. Growth performance is known to reduce after castration, particularly as the age of the animal increases (Bretschneider 2005). The only treatment differences found in ADG were seen in a short window and favored the CON treatment, but the SAA on either treatment had improved gain for the entirety of the receiving period.

Even though growth performance did not improve, morbidity incidence associated with BRD decreased with MBAS administration at receiving. The morbidity results demonstrate that MBAS administration has the potential to alleviate stress that is associated with castration, which may have been significant with larger sample sizes. Similarly, a 42-d study conducted by Schubach et al. (2020) administered MBAS to beef calves at weaning and reported an improvement in humoral immunity to parainfluenza type 3 virus (PI3), bovine respiratory syncytial virus (BRSV), and BVDV. However, a more recent feedlot receiving study concluded that MBAS did not improve calf growth or BRD incidence, but did reduce mortality (Pickett et al. 2024). The short window of greater ADG in CON calves could be due to compensatory gain following respiratory distress. The average first day of treatment for BRD occurred within the first week of arrival. The current study also showed that sex had a greater effect on morbidity rates associated with BRD, which is likely due to the stress associated with castration at the start of the receiving trial.

The weaning process is one of the most stressful events within the beef production cycle and is known to stimulate adrenocortical and inflammatory responses that negatively impact overall cattle performance (Cooke 2017). While haptoglobin concentrations did not differ between MBAS and CON treatments, there was a tendency for an interaction of treatment and sex, with overall haptoglobin concentrations being the greatest in RCM on the CON treatment. Haptoglobin concentrations generally spike within 48 to 72 h after an injury/stress in cattle (Lomborg et al. 2008), which was observed in this study on d 3. The lack of positive main treatment effects on serum haptoglobin was unexpected due to the known association between this acute-phase protein metabolite with stress-related responses and inflammation (Cooke 2017; Fonseca et al. 2021).

The chute exit velocities recorded in this study were greater for SAA that did not receive MBAS administration. A greater exit velocity generally correlates with an animal that is undergoing stress, demonstrating that the CON steers were undergoing more stress than the MBAS steers. Agitation or aggressive-like behaviors expressed by cattle in response to human interaction can be attributed to their inability to cope with the situation and can be classified as a psychological stress reaction (Cooke 2014). Again, there was an effect of sex on CEV recordings, which showed that SAA were likely more stressed and had greater overall velocities than RCM.

To observe cattle behavior, the number of standing and lying bouts, bout duration, and the proportion of time spent both standing and lying were analyzed. The tendency for an interaction of treatment and ACS on the proportion of time spent standing in the current study suggests that castration might have caused discomfort. The number of standing bouts decreased for all calves similarly over the 7-d period. Increased activity on day 1 could have been associated with greater exploration of the pen and, thus, less fear of the novel environment. Schubach et al. (2020) conducted a similar receiving study and conducted live behavioral observations and reported MBAS increased activity on d 1, which was demonstrated in the overall performance of social and play behaviors exhibited. Lying behavior is a relaxed posture in cattle (Van Erp-van der Kooji et al. 2019). Overall, the expected results for standing and lying postures are not supported in the current study.

Behavior was assessed to determine if MBAS administration reduced agitation in the chute during handling; however, there were no effects observed. It was hypothesized that MBAS would have a calming effect on cattle temperament, as previous studies have linked its use with reduced adrenocortical and acute-phase protein responses in growing cattle (Francisco et al. 2012; Cooke et al. 2019). However, the current study did not observe effects of MBAS administration in either SAA or RCM.

To conclude, MBAS administration to high-risk calves did not improve overall growth performance within this 42-d receiving period but did decrease BRD incidence in RCM calves. A recent study reported that MBAS is only effective for up to 14 d (Pickett et al. 2024), which could potentially explain the day effects observed. The current study observed effects consistent with this timeframe, suggesting that MBAS may have a limited window of efficacy. Overall, data produced from this 42-d backgrounding study encourages further investigation on the effect MBAS could have to alleviate negative impacts of castration in weaned beef calves.

## Supplementary Material

txag038_Supplementary_Data

## List of abbreviations

ADG: Average daily gain
ACS: Arrival castration status
BRD: Bovine respiratory disease
CAS: Clinical attitude score
CEV: Chute exit velocity
CLARA: Clustering large applications
CON: Control
DM: Dry matter
ELISA: Enzyme-linked immunosorbent assay
MBAS: Maternal bovine appeasing substance
PBS: Phosphate-buffered solution
PI-BVDV: Persistent infection with bovine viral diarrhea virus
RCM: Recently castrated males
RIA: Radioimmunoassay
SAA: Steers at arrival
