# Supplementary material for: Effects of maternal appeasing substance and castration status on growth performance and health in newly received, high-risk beef calves
Source: Transl Anim Sci. 2026 Mar 27;10:txag038. doi: 10.1093/tas/txag038 (PMC13071398; doi:10.1093/tas/txag038)
Supplement: txag038_Supplementary_Data [file txag038_supplementary_data.docx]

**SUPPLEMENTAL TABLES**

**Supplementary Table 1.** Ethogram of behavior observations used in the chute.

| **Behavior** | **Definition** |
| --- | --- |
| Front end down | Chest in contact with the floor |
| Head tossing | Rapid head movements |
| Nasal discharge or salivation | Visible strings of snot or saliva handing or dripping |
| Vocalizing | Any noise made by the calf’s vocal cords |
| Whites of eyes visible | A significant amount of whites of the eyes visible in both eyes |
| Jumping/Thrashing/Pushing | Throwing of body forward, upward, or backwards in the chute |

**Supplementary Table 2.** Ethogram of behavior observations used to categorize chute scores.

| **Chute Score** | **Definition** |
| --- | --- |
| 1 | No movement |
| 2 | Restless shifting |
| 3 | Squirming and occasional shaking of chute |
| 4 | Continuous vigorous shaking of chute |
| 5 | Rearing, twisting body, violently struggling |

**Supplementary Table 3.** Effect of maternal bovine appeasing substance (MBAS) and arrival castration status (ACS) on chute scores of beef calves during a 42-d receiving study.

| **Item** | **MBAS** | | **CON** | | **SEM** | ***P* - value** | | | |
| --- | --- | --- | --- | --- | --- | --- | --- | --- | --- |
|  | **RCM^1^** | **SAA^2^** | **RCM** | **SAA** |  | **ACS** | **Treatment** | **ACS × Treatment** | **ACS × Treatment × Day** |
| Chute score^3^ |  |  |  |  |  |  |  |  | 0.4029 |
| d 3 | 2.50 | 2.71 | 2.08 | 2.94 | 0.23 | 0.0126 | 0.6565 | 0.1220 |  |
| d 14 | 1.86 | 2.18 | 2.00 | 2.43 | 0.20 | 0.0413 | 0.2750 | 0.7311 |  |
| d 28 | 2.18 | 2.47 | 2.54 | 2.31 | 0.23 | 0.8888 | 0.6361 | 0.2265 |  |
| d 42 | 1.86 | 2.06 | 1.92 | 2.31 | 0.21 | 0.1341 | 0.4344 | 0.6087 |  |

^1^RCM = recently castrated males.

^2^SAA = Steers at arrival.

^1^Chute score was determined based on a score from 1 to 5 (1 = no movement, 2 = restless shifting, 3 = squirming occasional shaking of chute, 4 = continuous vigorous shaking of chute, 5 = rearing, twisting body, violently struggling
